# Bioconversion Potential of Agro-Industrial Byproducts by *Tenebrio molitor*—Long-Term Results

**DOI:** 10.3390/insects13090810

**Published:** 2022-09-06

**Authors:** Anna Bordiean, Michał Krzyżaniak, Mariusz Jerzy Stolarski

**Affiliations:** Department of Genetics, Plant Breeding and Bioresource Engineering, Faculty of Agriculture and Forestry, University of Warmia and Mazury in Olsztyn, 10-724 Olsztyn, Poland

**Keywords:** edible insects, mealworms, small livestock, novel food, bioconversion

## Abstract

**Simple Summary:**

Due to the forecasted overpopulation of the globe in the coming decades, the possibility of using nutrients (proteins, fats, vitamins) from sustainable sources such as insects has been discussed extensively. Although insects have been widely consumed in Asian and Latin American countries since ancient times, they are a novel food on the European continent. The first insect-based product that was allowed on the European market in early 2021 was powdered yellow mealworm (*Tenebrio molitor*), and later, in December 2021, frozen and dried mealworms. Agricultural byproducts are a valuable source of nutrients that can serve as a feed for insect rearing, contributing to better bioconversion of nutrients and promoting insects as a safe protein source. The present study has evaluated the bioconversion potential of yellow mealworm larvae grown on 13 diets composed of different agro-industrial byproducts. During the experiment, the larval weight gain and feed consumption were evaluated to calculate the feed conversion ratio and efficiency of ingested feed, as well as the larval development time. The results show that the best mixes for insect rearing were those that contained rapeseed meal and chicken feed, as they significantly improved the feed conversion ratio and efficiency of ingested larvae feed.

**Abstract:**

The aim of this study was to compare the growth performance, feed conversion ratio (FCR), and efficiency of ingested feed (ECI) by larvae of the *Tenebrio molitor* beetle. The growth and development potential of the yellow mealworm was evaluated by using chicken feed (CF), rapeseed meal (RM), wheat bran (WB), and willowleaf sunflower (WS) to obtain inexpensive and various sources of feed. The results showed that the fastest growth with shorter larval development time (74.7 days) was found for insects fed a WB 100 diet. Regarding the final fresh weight of larvae, the highest values were found for larvae grown on WB diets. However, the lowest FCR (1.53 to 1.59) was found for larvae fed RM diets mixed with CF and on the control diet (CF 100). In most cases, it was found that byproduct diets mixed with CF improve the ECI of mealworms, thus contributing to an efficient bioconversion of byproducts into edible sources of nutrients. Thus, except for pure WS as a feed, these byproducts can be used to grow yellow mealworm larvae and may be added to the versatile list of substrates and feed used by small- and large-scale insect producers.

## 1. Introduction

The circular economy concept involves the reduction of waste through reuse, repair, reconditioning, rethinking, and recycling, thus extending the life cycle of products. In this way, the linear economy (in which the product is made from resources, purchased, used, and then disposed of) has led to the generation of a significant amount of waste each year, which affects the quality of the environment and contributes to the depletion of resources. Thus, by 2050, a 70% increase of the currently produced annual waste is expected (2.01 billion tonnes of waste in 2016 and 3.4 billion tonnes in 2050) [1] as a result of the predicted population growth. Researchers are seeking solutions to reduce the reckless depletion of resources and are searching for new sustainable resources. One solution is to intensify all economic branches to a much higher level. Two important pillars in tackling future challenges are the food industry and agriculture. Although about one third of all food produced annually is thrown away or wasted [2], that waste does not come from food surpluses alone, as there is still famine around the world [3]. The wastage is caused mostly by insufficient knowledge, technical limitations in harvesting, and defective processing up to consumer steps, as well as ignorance regarding the efficient management of resources. Around 44% of the waste generated is from food and green waste [1]. In this way, around 30% of cereals, 40–50% of root crops, fruits, and vegetables, 20% of oilseeds, meat, and dairy products, and 35% of fish [4] are lost. Thus, the diverse types of agro-industrial and food waste, before reaching landfill or being used for energy purposes, should be investigated to recover their nutrients. In the context of a circular economy, a solution for nutrient recovery could be the use of insects for efficient bioconversion. One of the most extensively investigated insects in Europe is the *Tenebrio molitor* beetle, known as the yellow mealworm, and its larvae [5]. Although this species is often associated with stored grain [6], the mealworm’s gut is capable of digesting a variety of substrates or byproducts, such as waste from bakeries (bread remains, cookie remains), brewery yeasts, potato peelings, spent grains, beet molasses [7,8], spirit distillers’ grains, highly denatured soybean meal, mushroom spent corn stover [9,10], agricultural byproducts from the seed cleaning process of cereals (triticale, barley, durum wheat, and oat), and legumes (vetch, pea, lupin, lentil, lucerne and broad bean) [11], as well as industrial byproducts (chicken feed, wheat and rye brans, rapeseed meal and cake, flax cake, and *Silybum marianum* cake) [12,13]. In this way, mealworm larvae contribute to the efficient bioconversion of valuable nutrients from byproducts and waste, thus becoming a nutritious feed for other livestock (i.e., chicken, fish) [14,15]. The use of residues as feed has obvious economic or environmental effects from the viewpoint of circular bioeconomy. In insect farming, as in other branches of animal rearing, it will also be important to achieve high animal growth and a low value of feed conversion ratio (FCR).

This research is a continuation of a previous study which did not obtain conclusive results on the FCR of the different diets used in yellow mealworm farming [12]. However, some of the examined features (in particular FCR) showed only slight statistical variability. Therefore, it was decided to conduct two more trials in order to obtain more data and more reliable results. Hence, the investigations were supplemented with additional results, and additional insect features were examined, e.g., pupation time.

The objective of this study was to investigate the growth performance, survival, pupation time, feed conversion ratio (FCR), and efficiency of ingested feed (ECI) of yellow mealworm larvae on different agro-industrial byproducts.

## 2. Materials and Methods

### 2.1. Insect Samples/Yellow Mealworm Colony

The starter colony of *T. molitor* larvae (L. Coleoptera: Tenebrionidae) used in this study was obtained from a commercial supplier (CRICKETSFARM, Motycz-Józefin, Poland). The purchased mealworms (approximately 11th–12th instar) were reared to obtain an adult colony. The insects were kept in a rearing facility under monitored environmental parameters (28 °C, 55–60% RH, 12 h photoperiod). They were fed ad libitum with milled 3 mm chicken feed and supplied with carrot slices three times per week. The insects were kept in plastic containers (35 × 23 × 13 cm) with aeration holes on the sides, closed with a lid. A few weeks later, pupae were obtained. After another 6–7 days, the first adult beetles emerged. They were moved to “an adult box”, constructed with a mosquito net as a bottom that allowed eggs and small feed particles to fall into an egg collection box. The beetles were collected until their population was large enough to obtain a high number of eggs in a 3–4-day period. Hatching was estimated to occur 7–10 days after the egg collection period. Newly emerged larvae were fed ad libitum chicken feed and fresh carrot for four weeks. This allowed larvae to grow to a 3rd–4th instar that enabled their safe and easy collection for the main experiment. Fifty larvae were placed in a experimental box (22 × 13 × 5 cm, approx. one larva per 5.7 cm^2^) with aeration holes on the sides, covered by a lid.

### 2.2. Diet Preparation

Prior to the experiment, various cheap agricultural/industrial residues were purchased from commercial companies in Poland. The chicken feed (used as a control) was composed of corn kernels, wheat gluten feed, wheat, soybean meal (with GMO), calcium carbonate, vegetable oil, sodium chloride and rapeseed meal. The only exception was willowleaf sunflower (*Helianthus salicifolius*) biomass, which was obtained from an experiment conducted in the Department of Genetics, Plant Breeding and Bioresource Engineering of the University of Warmia and Mazury in Olsztyn, Poland. The experimental diets were composed of (1) chicken feed, used as a control feed (CF), (2) rapeseed meal (RM), (3) wheat bran (WB) and (4) willowleaf sunflower (WS) biomass originating from residues after supercritical CO_2_ extraction of high-value active compounds [16].

All feeds were comminuted through a sieve with a 3 mm diameter. The obtained feedstocks were sieved through a 300 μm sieve to remove the smallest particles with sizes similar to the larva faeces, which could be confused as frass. Table 1 presents the approximate composition of experimental diets based on raw ingredients. Proximate analysis of experimental diets included ash and fiber content, crude fat, protein content and nitrogen-free extract (NFE). Ash content was measured by using an automatic ELTRA TGA-THERMOSTEP analyser (Neuss, Germany) according to the PN-EN ISO 16948:2015-07 and PN-EN ISO 16994:2016-10, and fiber was analysed according to the PN-EN ISO 13906:2009, by using ANKOM A200 (Macedon, NY, USA) respectively. Protein content was measured using the Kjeldahl method, and crude fat was determined using Soxhlet extraction with petroleum ether as a solvent. The NFE estimates nonfibrous carbohydrates, such as soluble mono- and oligosaccharides and starches. The NFE was calculated according to Equation (1):

NFE (% dry mass) = 100% − [P (% d.m.) + CF (% d.m.) + CFb (% d.m.) + Ash (% d.m.)],(1)

where P is crude protein content, CF crude fat, and CFb is the crude fiber content in the diets. Subsequently, diets were mixed in different weight proportions with the chicken feed: 0, 25, 50, and 75%. Thus, 13 mixes of feed were obtained. Diets were stored at −20 °C until the beginning of the experiment. For reliable data collection, the full experiment included three separate trials, each conducted in 3 replicates (*n* = 9). Therefore, 150 larvae were simultaneously tested on each diet per trial (50 per box) and 450 in total for three trials. Each larvae box was provided with 3.1 g of experimental diet at the beginning of each experimental week. Subsequently, the feed amount was adjusted following the larvae’s growth.

### 2.3. Larval Growth and Development Measurements (Feeding Trial)

The larvae weight gain (LWT), individual larvae weight (LW), and feed consumption were calculated as previously described [12]. The larvae were screened for frass every week. Subsequently, the weight of 20 randomly chosen larvae from each box was registered. The larvae were separated by sieves to separate them from the frass and they were then collected manually with paper strips (to protect the insects from damage). Therefore, following this measurement, the individual weight (LW) of larvae and larvae weight gain (LWT) (mg fresh matter (f.m.)) were calculated. All larvae were returned to the same box for further measurements. The final fresh and dry weights of larvae grown on WS 100 in this study are based on the second and third trials, as the harvesting on the first trial was terminated after the first pupation in the whole trial and larvae fed on WS 100 were still far from the pre-pupa stage [12].

The feed consumption in the dry and wet forms was assessed by subtracting the remaining feed mass from the feed provided at the start of each experimental week. The same was done for consumed carrots.

The first trial was terminated when the first pupation occurred, regardless of a diet [12]. The pupation time per diet was recorded in the second and third trials. The pupation time in each box was recorded, and the larvae development time (larval stage) was considered to have been completed when at least one pupa was detected per diet.

Based on these parameters, the feed conversion ratio (FCR) and efficiency of conversion of ingested feed (ECI) were calculated. The FCR was calculated by dividing the total mean of individual larvae consumption by the total mean of individual weight gain (as described by Miech et al. [17]) and calculated on a wet basis:

FCR = weight of ingested food/weight gained,(2)

The ECI values were recorded at the end of the experiment. The ECI was calculated based on Waldbauer [18] by dividing the final weight of larvae by the weight of ingested feed, and the result was expressed on the dry matter basis of feed and insects:

ECI = (final weight/weight of ingested feed) × 100 (%),(3)

### 2.4. Statistical Analysis

A statistical analysis was performed with Statistica 13 software (Tibco, Palo Alto, CA, USA). Experimental data, such as individual fresh weight, individual dry weight, survival, FCR and ECI, were subjected to a one-way ANOVA analysis. Homogeneous groups for all features were determined using Tukey’s (HSD) multiple tests at α = 0.05. The results from the larval stage were not included in the ANOVA statistics because the data (being the results of two repetitions/trials) were considered insufficient to be processed statistically.

## 3. Results and Discussion

The results of a one-way ANOVA analysis (Table 2) proved that the studied diets influenced the final fresh and dry weight of larvae (f.m.), as well as the FCR and ECI. However, the larvae survival rate was the same among all of the tested diets.

The survival of mealworms on the tested diets (Table 3) was high, between 92.2% and 97.7% for larvae grown on WB 100 and WS 25/CF 75, respectively. Owing to the low density of mealworms in a box (50 larvae per box) and the sufficient amount of feed, the mortality was low. The present results on mortality are very low compared to the literature [19], where mortality was much higher among larvae fed with wheat bran (mortality 45%) and slightly lower for insects grown on ware potatoes (25%), but the highest mortality (75%) occurred for larvae fed on polystyrene foam. In another study on yellow mealworms fed different diets, the survival rate ranged between 71% and 92% for larvae fed on control diets (commercial diet) and HPLS (high-protein and low-starch diet), respectively [8]. The use of some diets with low protein and high fat significantly reduces the survival of mealworm larvae (15% and 19% for LPHF) [7]. However, a low-fat diet in the same study improved the survival of larvae up to 52% and 80%. Silva et al. [20] showed that the survival of insects ranged between 66.8% and 81.3% for larvae grown on diets containing 50% and 25% of poultry litter, respectively. In other research, the authors found that mealworm larvae survival might decrease to 87% in cases of increased care manipulations (weekly frass removal and adding new fresh feed) compared to the batches where these manipulations were minimized, where it reached 97.4% [21]. The type of water supply to mealworms also plays an important role by shortening the development time and improving the survival of larvae [7]. Thus, the larvae raised for four weeks on wheat bran (as a control) and wheat bran supplemented with various vegetables (fresh carrots, red cabbage leaves and orange) had high survival rates (91.7%, 89.3%, 92.5% and 89.7%, respectively) [22] compared with larvae reared on different agricultural byproducts obtained after the cleaning process of the cereals: triticale, barley, durum wheat and oat (64–84%) and legumes: vetch, lupin, pea, lentil, lucerne and broad bean (24–52%) [11]. In the current study, sporadic cases of cannibalism were registered between larvae. However, it cannot be determined whether acts of cannibalism were the cause of death or if they occurred after an insect’s death. It is assumed that the lack of some nutrients or hunger can force larvae to resort to cannibalism or to eating dead larvae or exoskeletons [23]. However, in some cases, weakened larvae may be less aggressive against other individuals (insects fed with polystyrene foam) [19]. A reduction in larval aggression (and, therefore, a higher survival rate) was observed in the present study, especially for larvae fed on WS diets. Survival can also be influenced by temperature. In a recent study [24], it was found that temperature is an important factor in the survival of mealworm larvae; around 92% of larvae survived at 20 °C, compared to the higher survival at 97% and 96.7% for mealworms maintained at 25 °C and 30 °C, respectively. Moreover, based on other results [25], the authors state that a temperature below 22 °C and above 35 °C reduces survival.

### 3.1. Larvae Development Time

The larvae fed on WB 25/CF 75 started to pupate after 73 days (Table 3). The longest larval period (115 days) was recorded for mealworms fed on the WS 100 diet. The development time from hatchling to pupation of mealworm larvae grown on CF (control diet) was 76.8 days. The duration of the larval stage depends on a number of factors, including the temperature, the feed used, and its nutritional characteristics. The larval stage of mealworms fed on 50:50 distillers dried grain mixed with wheat bran, and on 100% wheat bran lasts for around 43.61 and 49.77 days, respectively [26]. In another study [9], pupation occurred 60 days after hatching among mealworms fed on wheat bran, corn stover soybean meal and distiller grains. Based on other studies [7,8,11,20], it can be concluded that the larval stage duration varies and depends mostly on the composition of diets and ratios between the nutrients. The larval stage could last longer for larvae grown on low-protein and high-starch diets (up 225 days) [8] or low-protein and low-fat diets (up to 227 days) [7]. Diets with a higher protein content shorten the larval stage regardless of the ratio of fat (HPHF—88 days and HPLF—83 days) [7]. Breeding mealworm larvae under dry conditions (50% RH and without any water supplementing) lengthened the larval stage to 170–229 days [27]. Moreover, depending on the origins of mealworm strains, some could be more resistant (Turkish strain) when growing under dry conditions and have a shorter larval stage than other strains [27]. Therefore, longer larval development time until pupation involves extending the energy resources (such as heat, light, and handling) to obtain larvae ready for consumption or a colony of adult beetles. On the other hand, the literature indicates that prolongation of the larval stage may be caused by excessive or invasive care (increased handling) of yellow mealworm larva [21]. The temperature at which mealworms are raised influences the length of the larval stage. There was a significant difference between the larval stage duration for larvae reared at 20 °C (184.8 days) and those reared at 25 °C and 30 °C (138 and 136.1 days, respectively). It has also been found that the mealworm larval stage was generally shorter under short days (8 h of light) and 24 h of darkness [24].

### 3.2. Growth Performance

**Figure 1:**
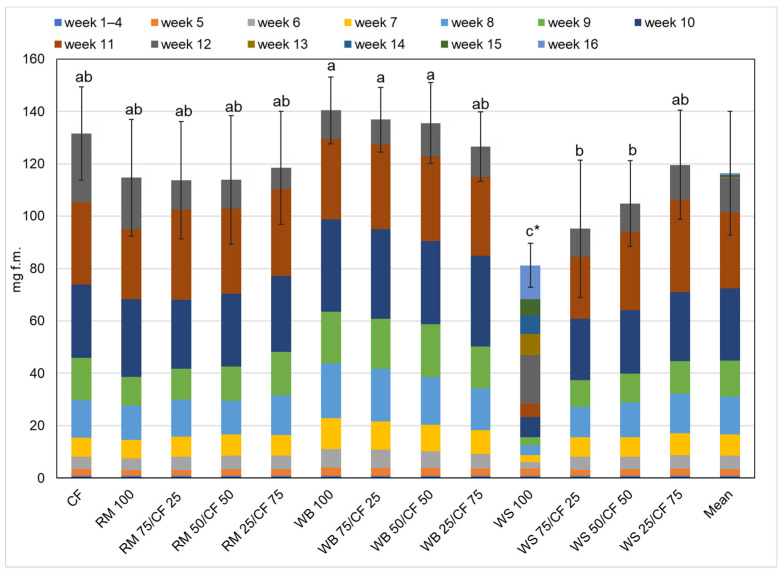
Individual fresh weight of yellow mealworm larvae depending on the week and the experimental diet. a,b,c…—letters indicate that values are statistically different (Tukey’s test at *p* < 0.05), (* the mean of 2 trials).

Although the length of the larval stage is an important factor in insect farming, the final weight of the harvested larvae plays a major role as well. The significantly highest final fresh larval weight (Figure 1) was found for mealworms fed with WB 100, WB 75/CF 25, and WB 50/CF 50 (140.4, 136.9, and 135.5 mg f.m., respectively). The fresh weight of mealworms fed on the control diet was remarkably similar (131.5 mg f.m. per larvae). The final fresh larval weight was not significantly different between the diets containing RM, and ranged between 113.6 and 118.6 mg f.m., for RM 75/CF 25 and RM 25/CF 75, respectively. Therefore, it can be concluded that diets with RM (pure) or mixed with CF can be used in any proportion with respect to the final fresh larval weight. The same situation was found in a preliminary trial, in which the weights differed slightly between larvae grown on RM diets [12]. In the case of WS diets, it was observed that a higher CF ratio led to the higher weight of mealworms. The lowest larval fresh weight was determined for insects grown on WS 100 diet (76.6 mg f.m.).

**Figure 2:**
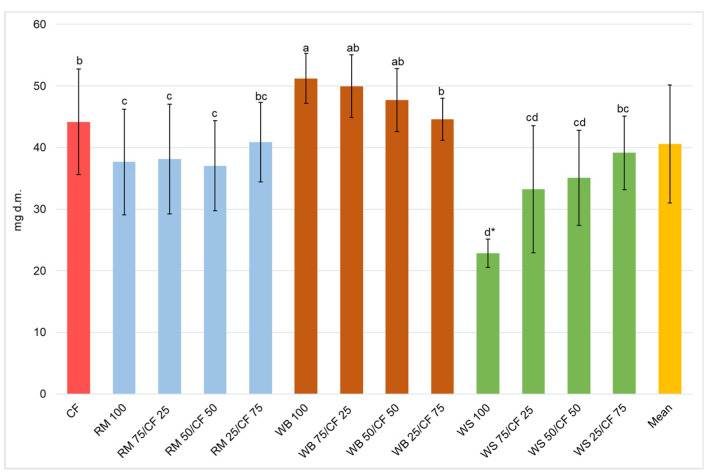
Individual final dry mass/weight of yellow mealworm larvae depending on the experimental diet. a,b,c…—letters indicate that values are statistically different (Tukey’s test at *p* < 0.05), (* the mean of 2 trials).

The individual dry weight of larva fed on different diets was 40.6 mg d.m. on average (Figure 2). As in the case of fresh weight, the significantly highest dry weight of larvae (51.2 mg d.m.) was found for insects fed on WB 100 diet, being included in homogeneous group a. The diets with a high ratio of WB (WB 75/CF 25 and WB 50/CF 50) resulting in significantly lower dry weight (50.0 and 47.7 mg d.m., respectively) were included in an intermediate homogeneous group ab. In terms of the dry weight of larvae, the control (CF) and WB 25/CF 75 diets were included in another homogeneous group (b), in which the larvae weighed around 44 mg d.m. The significantly lowest individual dry weight (22.8 mg d.m.) was found for larvae grown on WS 100 diet.

The final fresh weight of larvae found in the literature differed from the data in the current study, depending on the type of feed used for mealworms, the type of water supplementation or its lack, the origin of strains, and the rearing temperature. Therefore, the final fresh weight of the pre-pupa larvae depends on the quality and nutritional characteristics of the feed used for the mealworm breeding [8]. Depending on the mealworm strains, larvae may have a different fresh final weight (87 to 154 mg, USA strain and German strain, respectively) [28]. The water source, provided in the form of a vegetable or fruit, is also important. Thus, larvae fed with wheat bran and different vegetables were heavier than in the present study (150 mg per larvae) and compared to larvae fed without a water source (only on wheat bran) (around 110 mg per larvae) [22]. In other studies, deprivation of water sources of mealworms reduces the final fresh weight of larvae (max. to 78 mg per larvae) [27]. Other researchers [21,28] also found that a longer larval development period of yellow mealworm contributes to a higher final fresh weight (up to 137 mg). The optimum temperature for mealworm larvae contributes to a higher larval weight. In another study [24], it was found that larvae grown at 30 °C reached a higher larval weight (between ~150 and 160 mg) than those grown at 20 °C (max. ~120 mg).

Data on the final dry weight of mealworm larvae found in the literature range from 35.5 to 67 mg per larvae depending on diets (plant waste diet and mushrooms, spent corn stover diets, respectively) [9,29].

**Figure 3:**
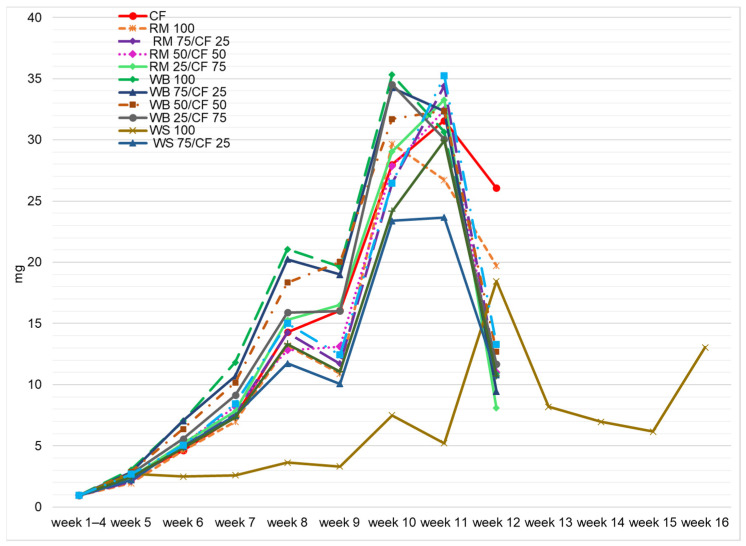
Individual larval weight gain (mg f.m.) depending on the type of diet and week of rearing.

In general, the larval weight gain (LWG) doubled weekly compared with the prior measurement week (Figure 3), except for the period from the 8th to 9th weeks, when LWG was slightly lower than in the previous weeks. Another important aspect is that mealworms prepare for pupation before the end of the larval stage (before the 10th and 11th weeks), in which they consume less feed. The literature provides similar observations about the growth rates of mealworms that slow down in the last 20 days of the larval period, except for those bred on wheat bran (control feed) [9]. Thus, it could be stated that the feeding of insects could be discontinued even one week earlier before pupation. With respect to insect breeding facilities, this aspect is relevant for production economics. However, this process is completely different, and LWG rates were lower in the case of mealworms fed on WS 100. The highest LWG was recorded in week 12, decreasing afterwards. The subsequent increase of LWG for larvae fed with WS 100 took place just before pupation. Since the consumption of feed lacking in nutritive properties makes larvae eat less, they will add less weight [30].

**Figure 4:**
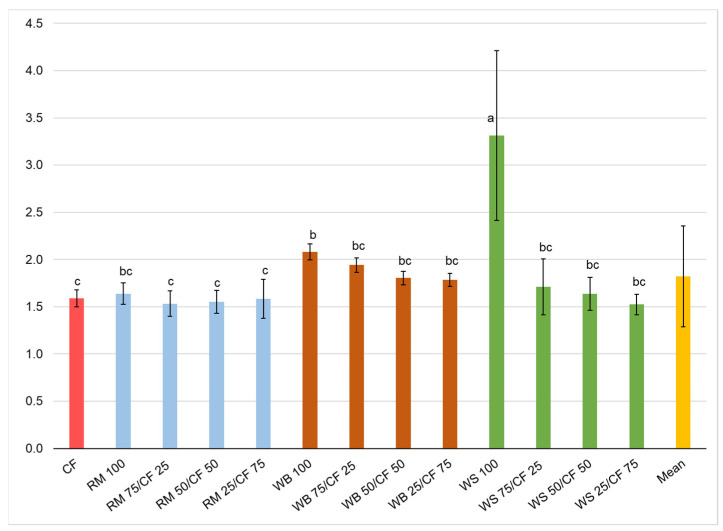
Mealworm feed conversion ratio (FCR). a,b,c…—letters indicate that values are statistically different (Tukey’s test at *p* < 0.05).

The FCR of larvae differed significantly between the tested diets (Figure 4). The significantly highest FCR (2.08) was recorded for insects fed with WS 100. Significantly the lowest FCR was found for insects fed with all RM/CF diets and on the sole CF diet, where it ranged from 1.53 to 1.59 for RM 75/CF 25 and CF 100, respectively. The FCR for mealworms fed on the remaining diets was slightly higher, falling into the intermediate homogeneous group bc and b (excluding WB 100 diet). Therefore, the type of feed used for mealworm rearing has an important impact on the development and growth performance of larvae. The current results suggest that the protein content of feed influenced the indicators of insect development. In the current study, the FCR of larvae grown on the highest protein content diets (RM diets) was the lowest compared with larvae grown on the diets with less protein (especially on WS 100 diet). The FCR differed significantly when agricultural byproducts of the cereal and legumes cleaning processes were evaluated as feed for mealworm larvae. The FCR ranged between 2.6 and 7.6 for larvae grown on cereal byproducts (triticale and durum wheat, respectively), and between 1.7 to 12.6 for larvae fed on legumes byproducts (pea and lucerne, respectively) [11]. The scientific literature discusses relationships between the protein content of a feed ingredient and the FCR; this aspect is carefully evaluated when formulating various diets from byproducts [7,8]. Data provided from the literature confirm that mealworm larvae grown on diets with a high protein content (32.7 and 39.1% d.m. CP) showed higher FCR than larvae grown on a low-protein-content diet (11.9% d.m. CP) (3.04, 2.62, and 6.05, respectively) [8]. It was found that the protein content of diets plays a key role in the nutrition and development of not only yellow mealworm larvae but also other species of mealworms (giant mealworm *Zophobas atratus* Fab., and lesser mealworm *Alphitobius diaperinus* Panzer) [8].

**Figure 5:**
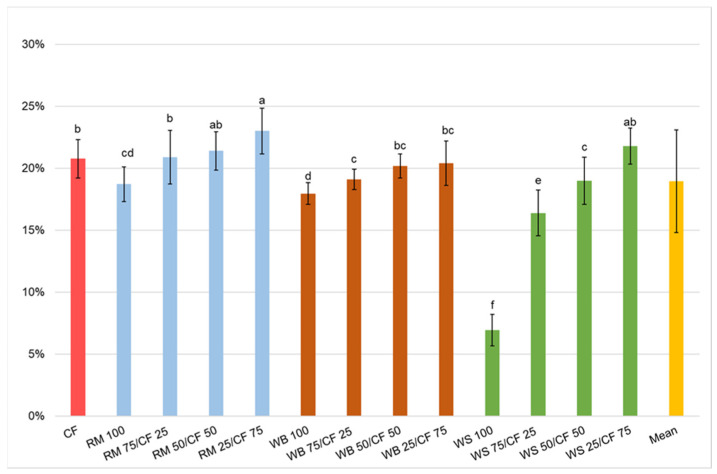
Mealworm efficiency of ingested feed (ECI) conversion. a,b,c…—letters indicate that values are statistically different (Tukey’s test at *p* < 0.05).

It is obvious that there is an inverse relationship between the FCR and ECI, also confirmed by the literature [8]. The ECI values were different between the mealworm grown on different diets (Figure 5). It was found that a higher ratio of CF in diets was correlated with higher ECI. The significantly lowest ECI (the worst) (6.9%) was recorded for mealworms grown on WS 100. Therefore, an inadequate or insufficiently nutritious diet will be ingested by insects in a larger amount but with lower efficiency, as could be deduced from the FCR and ECI values. This was especially observed in the case of mealworms fed on the WS 100 diet, in which both ECI and FCR values were unacceptable. Other researchers found that a higher fat content in diets entailed a higher ECI. Broekhoven et al. [8] recorded that the ECI for insects fed with diets with 5.5 and 5.8% d.m. fat was above 28% and was higher than for the control diet and low-protein high-starch diets (with 3.0 and 2.3% d.m. of fat, respectively). In those diets, the ECI equalled 18.96% and 16.76%, respectively.

The fat content of diets influences the FCR of mealworms as well. Thus, the FCR of larvae grown on low-fat diets (0.4 and 1.5% fat d.m.) and on high-fat diets (8.9 and 14.0% d.m. of TFA) was 4.1, 6.1, 3.8, and 5.3, respectively [7]. Besides protein and fat, there are other factors that influence the FCR, such as starch, strains of insects, and growing conditions (lack of moisture). High-starch diets contributed to the increased FCR for mealworm larvae [8]. The FCR can vary between mealworm strains. Rumbos et al. [28] found that the FCR of mealworm larvae growing on the same diet and under the same condition varied between 2 and 2.6 (Italian and German strains, respectively) [28]. The ECI also depends on strains of larvae. Thus, the best ECI (21.0, 19.9 and 19.7%, respectively) was for Italian, Turkish and Greek strains of mealworms [28]. The strain type also influences the FCR and ECI when there is a shortage of moisture and water in a feed (6.7 and 10.2 for FCR and 6.3 and 4.3% for ECI, for Turkish and Greek strains, respectively) [27].

## 4. Conclusions

The diets from byproducts evaluated in this study had a different impact on the tested features of yellow mealworm larvae. The results showed that larvae survival was high and not influenced by the experimental diets. The length of the larval period was typically from 73 to 82 days (for WB 25/CF 75 and WS 75/CF 25, respectively), except for larvae fed on WS100 (115 days). The highest final fresh and dry weight of harvested larvae was found for those grown on WB diets. The results confirm that a diet based only on WS was not sufficient nutritionally for their efficient growth and resulted in a long larval stage, low larval fresh and dry weight, slow larval weight gain, and unfavourable FCR and ECI values. Adding CF into the diets significantly improved the FCR compared to the sole diets. The same tendency was observed for the ECI, where larvae used the feed more efficiently. Based on the results, it can be concluded that the most efficient, rational and balanced diets related to both FCR and ECI are those that contain RM mixed with CF. The current results also show that other feeds mixed with CF show satisfactory results by improving these indices. However, future studies should investigate the effect of diets on the quality of the next insect generations, especially the survival and development of larvae and pupae and beetle fertility.

## Figures and Tables

**Table 1:** Proximate composition of experimental diets.

	Diets	Ash (% d.m.)	Fibre Content (% d.m.)	Crude Fat (% d.m.)	Protein (% d.m.)	NFE (% d.m.)
1.	CF 100	12.89	3.23	2.38	20.11	61.39
2.	RM 100	7.39	11.12	1.78	35.52	44.19
3.	RM 75/CF 25	8.77	9.15	1.93	31.67	48.48
4.	RM 50/CF 50	10.14	7.18	2.08	27.82	52.78
5.	RM 25/CF 75	11.52	5.2	2.23	23.96	57.09
6.	WB 100	5.82	8.68	2.90	18.63	63.97
7.	WB 75/CF 25	7.59	7.32	2.77	19.00	63.32
8.	WB 50/CF 50	9.36	5.96	2.64	19.38	62.66
9.	WB 25/CF 75	11.12	4.59	2.51	19.74	62.04
10.	WS 100	9.49	22.64	0.76	10.56	56.55
11.	WS 75/CF 25	10.34	17.79	1.17	12.95	57.75
12.	WS 50/CF 50	11.19	12.94	1.57	15.34	58.96
13.	WS 25/CF 75	12.04	8.08	1.98	17.72	60.18

Abbreviations: CF—chicken feed, RM—rapeseed meal, WB—wheat bran, WS—willowleaf sunflower, NFE—nitrogen-free extract.

**Table 2:** Results of one-way ANOVA analysis.

Source of Variation	Survival	Dry Weight	Fresh Weight	FCR	ECI
df	12				
F	1.7	9.416	6.234	25.219	60.44
*p*	0.07	<0.001	<0.001	<0.001	<0.001

FCR—feed conversion ratio, ECI—efficiency of conversion of ingested feed.

**Table 3:** Survival and larval stage duration (from hatching to pupation) of larvae grown on different agro-industrial byproducts.

No.	Diet	Survival (%)	Larval Period (Days) *
1.	CF (control)	96.0 ± 2.6	76.8 ± 3.2
2.	RM 100	95.3 ± 2.8	78.5 ± 0.5
3.	RM 75/CF 25	96.9 ± 1.4	76.0 ± 1.0
4.	RM 50/CF 50	96.7 ± 2.4	76.0 ± 1.0
5.	RM 25/CF 75	96.6 ± 1.9	76.0 ± 1.0
6.	WB 100	92.0 ± 6.7	74.7 ± 0.3
7.	WB 75/CF 25	96.0 ± 3.7	76.0 ± 1.0
8.	WB 50/CF 50	95.8 ± 2.5	76.0 ± 1.0
9.	WB 25/CF 75	95.8 ± 2.3	73.0 ± 2.0
10.	WS 100	95.8 ± 3.2	115.0 ± 1.0
11.	WS 75/CF 25	96.7 ± 2.4	81.5 ± 4.5
12.	WS 50/CF 50	96.2 ± 1.5	76.5 ± 1.5
13.	WS 25/CF 75	97.7 ± 2.2	75.0 ± 0.0
	Mean	96.0 ± 3.1	79.3 ± 0.6

CF (chicken feed), RM (rapeseed meal), WB (wheat bran), WS (willowleaf sunflower); ±—standard deviation; * average of two trials.

## Data Availability

The datasets used and/or analyzed during the current study are available from the corresponding author on reasonable request.
